# CRISPR/Cas9-mediated knockout of the Vanin-1 gene in the Leghorn Male Hepatoma cell line and its effects on lipid metabolism

**DOI:** 10.5713/ab.23.0162

**Published:** 2023-11-01

**Authors:** Lu Xu, Zhongliang Wang, Shihao Liu, Zhiheng Wei, Jianfeng Yu, Jun Li, Jie Li, Wen Yao, Zhiliang Gu

**Affiliations:** 1 School of Biology and Food Engineering, Changshu Institute of Technology, Changshu, 215500, China; 2 College of Animal Science & Technology, Nanjing Agriculture University, Nanjing, 210000, China

**Keywords:** Chicken, CRISPR-Cas9, Liver Metabolism, RNA-seq, *VNN1*

## Abstract

**Objective:**

Vanin-1 (*VNN1*) is a pantetheinase that catalyses the hydrolysis of pantetheine to produce pantothenic acid and cysteamine. Our previous studies have shown that the *VNN1* is specifically expressed in chicken liver which negatively regulated by microRNA-122. However, the functions of the *VNN1* in lipid metabolism in chicken liver haven’t been elucidated.

**Methods:**

First, we detected the *VNN1* mRNA expression in 4-week chickens which were fasted 24 hours. Next, knocked out *VNN1* via CRISPR/Cas9 system in the chicken Leghorn Male Hepatoma cell line. Detected the lipid deposition via oil red staining and analysis the content of triglycerides (TG), low-density lipoprotein-C (LDL-C), and high-density lipoprotein-C (HDL-C) after *VNN1* knockout in Leghorn Male Hepatoma cell line. Then we captured various differentially expressed genes (DEGs) between *VNN1*-modified LMH cells and original LMH cells by RNA-seq.

**Results:**

Firstly, fasting-induced expression of *VNN1*. Meanwhile, we successfully used the CRISPR/Cas9 system to achieve targeted mutations of the *VNN1* in the chicken LMH cell line. Moreover, the expression level of *VNN1* mRNA in LMH-KO-*VNN1* cells decreased compared with that in the wild-type LMH cells (p<0.0001). Compared with control, lipid deposition was decreased after knockout *VNN1* via oil red staining, meanwhile, the contents of TG and LDL-C were significantly reduced, and the content of HDL-C was increased in LMH-KO-*VNN1* cells. Transcriptome sequencing showed that there were 1,335 DEGs between LMH-KO-*VNN1* cells and original LMH cells. Of these DEGs, 431 were upregulated, and 904 were downregulated. Gene ontology analyses of all DEGs showed that the lipid metabolism-related pathways, such as fatty acid biosynthesis and long-chain fatty acid biosynthesis, were enriched. KEGG pathway analyses showed that “lipid metabolism pathway”, “energy metabolism”, and “carbohydrate metabolism” were enriched. A total of 76 DEGs were involved in these pathways, of which 29 genes were upregulated (such as cytochrome P450 family 7 subfamily A member 1, ELOVL fatty acid elongase 2, and apolipoprotein A4) and 47 genes were downregulated (such as phosphoenolpyruvate carboxykinase 1) by *VNN1* knockout in the LMH cells.

**Conclusion:**

These results suggest that *VNN1* plays an important role in coordinating lipid metabolism in the chicken liver.

## INTRODUCTION

Over the past several decades, enhanced genetic selection has improved the growth rate and feed conversion rate of broiler chickens but has also led to excessive fat deposition, particularly in the abdominal cavity [1]. Abdominal fat deposition is one of the most prominent issues in poultry production. This issue can cause a series of physiological disorders, such as obesity, ascites and overall decline in immunity, and can also lead to an increase in the cost of farming, which negatively affects the profitability of agriculture [2,3]. Unlike mammals, the avian liver is the main organ for lipid synthesis and plays a more important role in lipid metabolism. In addition, chicken liver lipid metabolism and abdominal fat deposition are regulated by genetic factors to a certain extent [4]. Previous studies have shown that miR-122, a liver-enriched noncoding RNAs, plays an important role in liver lipid metabolism [5]. Using RNA-seq, our laboratory found that the Vanin-1 (*VNN1*) gene, which is expressed almost exclusively in chicken liver, was significantly upregulated by miR-122 knockdown [6,7].

*VNN1* is a type of pantetheinase that is primarily expressed in liver, kidney, heart, and gut. *VNN1* can catalyse the hydrolysis of pantetheine to produce pantothenic acid (vitamin B_5_) and cysteamine (a highly effective antioxidant) [8]. Among the three orthologous Vanin genes (i.e., *VNN1*, *VNN2*, and *VNN3*), VNN1 is the most prevalent and important isoform and is involved in inflammation, oxidative stress, and cell migration [9]. Recently, it has been revealed that *VNN1* is closely related to fatty acid metabolism. For example, Diepen et al [11] found that RNAi-induced *VNN1* knockdown in mice aggravated the accumulation of liver triglycerides (TGs). Similarly, rats treated with the VNN1 inhibitor RR6 had more severe liver TG accumulation in response to fasting. Several different research groups have indicated that peroxisome proliferator activated receptor alpha (PPARα) can induce a robust increase in *VNN1* expression in the mouse liver and strongly modulate plasma Vanin activity [10,11]. Our study also revealed that PPARα increased the transcriptional activity of *VNN1* through binding to the putative PPARα-binding site located in the −49/−31 region of the *VNN1* gene promoter [12]. In addition, a clinical study showed that the combination of *VNN1* and matrix metallopeptidase 9 (*MMP9*) may be used as a novel blood biomarker panel to discriminate between pancreatic cancer-associated diabetes and type 2 diabetes [13]. These findings indicate that *VNN1* may play a crucial role in regulating lipid metabolism. However, the functions of the *VNN1* gene in lipid metabolism in the chicken liver have not been fully elucidated. Therefore, it is necessary to conduct an in-depth study on the function of the *VNN1* gene in chicken liver lipid metabolism, thereby providing new insights into the mechanism of regulation within chicken lipid metabolism.

In recent years, reverse genetics methods have been widely used to study the roles of coding genes, and the rapid development of the clustered regularly interspaced short palindromic repeats (CRISPR)/CRISPR-associated 9 (Cas9) technology provides an unprecedented opportunity to study these roles. The CRISPR/Cas9 system can introduce DNA double-stranded breaks at specific sites of the target gene, which can result in the efficient generation of insertion or deletion mutations by nonhomologous end-joining repair [14]. Due to the resulting high efficiency and rapid assembly, CRISPR/Cas9 has been extensively and efficiently utilized to precisely modify the genomes of many eukaryotic cells and organisms [15,16], including several cell types of chickens, such as chicken embryonic fibroblasts UMNSAH-DF-1 [17], chicken primordial germ cells (PGCs) [18] and early chick embryos [19].

Based on the findings mentioned above, we aimed to investigate whether VNN1 is involved in chicken liver lipid metabolism. To this end, we used the CRISPR/Cas9 system to mediate chicken *VNN1* knockout in LMH cells to study its function in liver lipid metabolism. In this study, we successfully used the CRISPR/Cas9 system to efficiently achieve targeted mutations in chicken LMH cells, which provided evidence for the successful application of CRISPR/Cas9 technology in poultry. Furthermore, our RNA-seq study also validated the differential expression of many lipid metabolism genes and the enrichment of pathways involved in lipid metabolism due to knockout of the *VNN1* gene in LMH cells.

## MATERIALS AND METHODS

### Animals and experimental procedures

Four-week-old Arbor Acres commercial chickens were housed under standard conditions with free access to water and feed. Each chicken feed group were in one cage and the volume of the cage was 35×38×42 centimetres. For the fasting group, chickens were fasted for 24 h, and for the refeeding group, the chickens were refed for 2 h after 24 h of fasting. The chickens used in the experiments were reared and euthanized with the approval of the Animal Welfare and Research Ethics Committee of the Changshu Institute of Technology (Permit number: EAWEC1710). Livers were immediately dissected, snap-frozen in liquid nitrogen, and stored at −80°C until further processing.

### Cells and cell culture

The chicken LMH cell line (ATCC) has well-differentiated morphological and biochemical characteristics and has been widely used as a good cell model for studying chicken liver lipid metabolism. Cells were maintained in Waymouth’s medium (Gibco, New York, NY, USA) containing 10% foetal bovine serum (Gibco, USA) and 100 U/mL penicillin/streptomycin in a humidified incubator (37°C, 5% CO_2_).

### Construction of the guide RNA expression vector

SgRNA targeting the chicken *VNN1* gene was designed using an online program (http://crispor.tefor.net) based on the 131 bp exon 2 sequence (GenBank: NM_001039288). The schematic structure of the chicken *VNN1* gene and a diagram of the sgRNA design are shown in Figure 2A. Three complementary sgRNA oligo DNAs (Table 1) were synthesized, annealed and then subcloned into a linearized pX330 vector (Addgene, Watertown, MA, USA). The pX330 plasmid map is shown in Supplementary Figure S1. These constructs were confirmed through sequence analysis.

### Cell transfection, T7 endonuclease I assay and thymine-adenine clone sequence

LMH cells (1.5×10^5^ cells/well) were plated in 24-well plates for 24 h and grown to ~70% confluence in antibiotic-free medium before transfection. Five hundred nanogram of the three knockout plasmids (pX330-*VNN1*-sgRNA1#, pX330-*VNN1*-sgRNA2# and pX330-*VNN1*-sgRNA3#) and negative control (ddH_2_O) were separately transfected into the LMH cells using X-tremeGENE9 DNA Transfection Reagent (Roche, Basel, Switzerland) at a transfection reagent/DNA ratio of 3:1. The LMH cells were treated with 2 μg/mL puromycin (Sigma-Aldrich, Rockville, MD, USA) after 48 h of transfection to enrich for pX330-transfected cells. The remaining LMH cells were then transferred to normal medium and allowed to proliferate for genomic DNA sequence analysis and the enrichment of positive cells. Genomic DNA of the cells was extracted for the T7 endonuclease I assay (T7E1) assay. Briefly, an approximately 397-bp fragment including the target site was amplified from extracted DNA using primers (Supplementary Table S1) PCR. The PCR products were denatured and annealed to generate heteroduplexes in NEB buffer 2 (NEB, Ipswich, MA, USA) using the following PCR programme: 95°C for 5 min, 95°C to 85°C at −2°C/s, 85°C to 25°C at −0.3°C/s, hold at 16°C for 2 min, and a final step at 4°C. After reannealing, the products were treated with 0.5 μL T7EI (NEB, USA) and incubated at 37°C for 30 min. Then, the digested PCR products were subjected to 2% agarose gel electrophoresis. Subsequently, thymine-adenine (TA) clones (n = 50) were selected for sequencing, to further validate the effect of knockout.

### Screening of positive subclonal cells

The positive cells obtained initially were further screened by 2 μg/mL puromycin until blank cells died. Subclonal cells (LMH-KO-*VNN1*) were then selected and cultured by limiting dilution. The cells were digested with trypsin, and the selected cells were collected and counted using trypan blue. The cell suspension was transferred to a graduated centrifuge tube and serially diluted to 10 cells /mL, after which 0.1 mL of the cells were added to a 96-well plate for culture. After the LMH-KO-*VNN1* cells reached the logarithmic phase, they were used for oil red staining, measurement of TG, low-density lipoprotein-C (LDL-C), and high-density lipoprotein-C (HDL-C) and RNA-seq experiments.

### Off-target analysis

Potential off-target sites for *VNN1*-sgRNA3# were predicted using an online site (http://crispor.tefor.net). Four potential off-target loci with the highest homology scores were selected for mutation analysis. The primers used for amplifying the off-target sites are listed in Supplementary Table S1. The PCR products were subjected to a T7E1 cleavage assay. Reference genomic sequences were obtained from Ensembl (WDR19, ENSGALG00000014064; FTO, ENSGALG0000 0041036; STK32A, ENSGALG00000029195; and ENSGALT 00000047140.1)

### Oil red staining

We modified the method of Xiong et al [20], and performed oil red O staining in this study. LMH cells and LMH-KO-*VNN1* cells were cultured at 3.0×10^5^ cells /mL in six-well plates at 5% CO_2_ and 37°C for 48 h, then fixed with 10% paraformaldehyde for 30 min and stained with oil red O for 30 min. After completion of staining, the oil red O dye was removed and washed with 60% isopropanol. Stained cells were observed and recorded with an inverted phase contrast microscope and photographed for comparison purposes. To further verify the lipid deposition after *VNN1* knockout, we used 100% isopropanol and incubated for 5 min with shaking to dissolve the oil red O dye. The solution was added to the microplate, and the optical density value at 510 nm was measured by a microplate reader, while the cellular protein was extracted to determine the protein concentration. According to the value of OD510/protein concentration, the difference of lipid deposition between LMH cells and LMH-KO-*VNN1* cells was determined.

### Detection of TG, LDL-C, and HDL-C content

LMH cells and LMH-KO-*VNN1* cells were plated at 3.0×10^5^ cells/mL in six-well plates, cultured at 5% CO_2_ and 37°C for 48 h. Then we trypsinized and collected cells with 0.25% trypsin, added lysate and ultrasonic disruption, and tested the content of TG, LDL-C, HDL-C in LMH cells and LMH-KO-*VNN1* cells via kit (Nanjing Institute of Bioengineering, Nanjing, China).

### RNA extraction and cDNA synthesis

Total RNA was isolated from LMH cells and liver tissue using RNAiso Plus (TaKaRa, Beijing, China) according to the manufacturer’s protocol and treated with RNase-free DNase. The concentrations of RNA were determined using a NanoDrop ND2000 spectrophotometer (Thermo Scientific, Wilmington, DE, USA). For reverse transcription, 0.5 μg of extracted RNA per sample was reverse transcribed using the PrimeScript RT reagent kit (TaKaRa, China) following the manufacturer’s instructions.

### Library preparation and transcriptome sequencing

Total RNA samples from *VNN1*-modified LMH cells were extracted using TRIzol reagent (Invitrogen, Waltham, MA, USA) and purified using the RNAclean kit (BioTeke, Beijing, China). Since the cells in the NC group were largely dead after the first round of puromycin screening, we cultured wild-type LMH cells as a control while px330-sgRNA3# was enriched for puromycin. Total RNA samples from wild-type LMH cells were extracted and purified in the same batch as *VNN1*-modified LMH cells. The amount of total RNA in all samples was at least 1 μg. RNA quality was verified using a Bioanalyzer 2100 (Agilent Technologies, Palo Alto, CA, USA). Total RNA was performed to isolate mRNA by poly-T oligo-attached magnetic beads and segregated into 200 to 300-bp fragments. First-strand cDNA was synthesized with hexahedron random primers and reverse transcriptase using RNA as a template, and second-strand cDNA was synthesized using the first strand cDNA as a template. After the library was constructed, library fragments were enriched by PCR amplification, and fragments of 300 to 400 bp were selected. The size and total concentration of cDNA libraries were assessed using the Agilent Bioanalyzer 2100 system and fluorescence quantitative, respectively. Transcriptome sequencing was performed by Shanghai Personal Biotechnology Co., Ltd. (Shanghai, China) based on the Illumina NextSeq500 sequencing platform using a 150-cycle paired-end sequencing strategy.

### Bioinformatic analysis

FastQC software was used for quality control. The Q20, Q30, and guanine-cytosine (GC) content of clean data were analysed, and all downstream analyses were performed using high-quality clean data. The high-quality clean data were mapped to the chicken reference genome (Gallus_gallus.GRCg6a.dna.toplevel.fa) using TopHat2 software. The original expression levels of each group of genes were analysed using the HTSeq program, and then the expression levels were normalized to FPKM (the reads per kilobase of the exon model per million mapped reads). Differential expression analysis between LMH wild-type cells and *VNN1*-modified LMH cells was performed using the DESeq package. Genes with an adjusted p-value<0.05 and fold change >2 in DESeq analysis were considered to be differentially expressed genes (DEGs). Gene ontology (GO) and Kyoto encyclopedia of genes and genomes (KEGG) enrichment analyses were performed to obtain GO functional entries that are significantly enriched for DEGs and metabolic pathways and signaling pathways in which the DEGs are primarily involved.

### Real-time quantitative polymerase chain reaction

cDNAs were amplified using SYBR Green PCR Master Mix (Applied Biosystems, Shanghai, China) in an ABI Prism 7500 sequence detection system (Applied Biosystems, China). The primers used for real-time polymerase chain reaction (PCR) are listed in Supplementary Table S1 and the positions of *VNN1* primer used for real-time quantitative polymerase chain reaction (RT-qPCR) were shown in Supplementary Figure S2. Quantitative PCR was performed in triplicate for each cDNA sample, and the results were normalized to endogenous actin mRNA. The data were analysed using the 2^−ΔCT^ method or the 2^−ΔΔCT^ method.

### Statistical analysis

All data are presented as the mean±standard error of the mean of at least 3 independent experiments. Statistical analyses were performed using Student’s t-test or one-way analysis of variance. A p-value <0.05 was considered to be statistically significant unless otherwise noted.

## RESULTS

### Fasting-induced expression of *VNN1* and related lipid metabolism genes in the chicken liver

**Figure 1:**
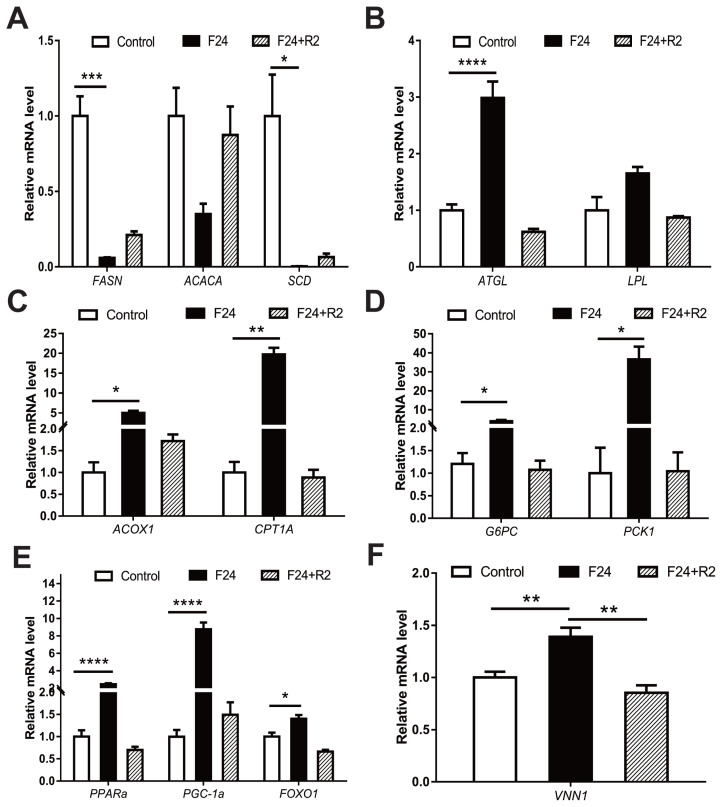
Expression patterns of metabolism-related genes in chicken livers in response to different feeding conditions. Fasting-refeeding cycles are an important model for studying energy metabolism. Thus, RT-qPCR analysis of *VNN1* and metabolism-related gene expression in the livers of chickens subjected to fasting (24 h) or fasting for 24 h followed by 2 h of refeeding. PCR was performed in duplicate with SYBR Green and specific primers for each gene (See Supplementary Table S1). β-Actin was used as a reference for normalization. Statistical significance is indicated as follows: * p<0.05, ** p<0.01, *** p<0.001, **** p<0.0001, n = 3–8. Control, fed ad libitum for 24 h; F24, fasted for 24 h after 24 h of ad libitum feeding; and F24+R2, refed for 2 h after 24 h of ad libitum feeding and 24 h of fasting. RT-qPCR, real-time quantitative polymerase chain reaction; *VNN1*, Vanin-1.

Fasting-refeeding cycles are an important model for studying energy metabolism. The mRNA expression levels of fatty acid synthase (*FASN*) and stearoyl-CoAdesaturase (*SCD*), the key genes controlling fatty acid synthesis in chicken livers, showed a significant decrease after 24 h of fasting. Subsequently, after 2 h of refeeding, the mRNA levels of the abovementioned fatty acid synthesis-related genes gradually increased (Figure 1A). Conversely, gene expression levels of genes that regulate TG degradation and fatty acid beta-oxidation (e.g., adipose triacylglyceride lipase [*ATGL*], acyl-CoA oxidase 1 [*ACOX1*], and carnitine palmitoyltransferase 1A [*CPT1A*]) significantly increased after fasting (Figure 1B and 1C). In addition, two gluconeogenesis genes (glucose-6-phosphatase catalytic subunit 1 [*G6PC*] and Phosphoenolpyruvate carboxykinase 1 [*PCK1*]) were significantly upregulated after fasting for 24 h (Figure 1D). Related studies have shown that hepatic energy metabolism is largely controlled by numerous transcription factors and coregulators. As shown in Figure 1E, some liver-enriched related transcription factors and transcriptional coactivators (e.g., *PPARα*, *PGC1α*, and forkhead box O1 [*FOXO1*]) in the livers of chickens were significantly upregulated after 24 h of fasting, which suggests that these transcription factors or coregulators may act as critical mediators to regulate hepatic glycolipid metabolism during the fasting period. Fasting triggers a complex series of adaptive metabolic reactions, including energy metabolism, which can result in specific changes in gene expression profiles. Adaptational changes in the abovementioned glycolipid metabolism-related genes and liver-enriched transcription factors are the most relevant examples. Interestingly, the expression level of the *VNN1* gene in the chicken liver also increased significantly after 24 h of fasting and returned to the original level after refeeding (Figure 1F). Moreover, VNN1 is a type of pantetheinase that can catalyse the hydrolysis of pantetheine to produce pantothenic acid (a precursor of CoA) [21]. Therefore, we speculated that VNN1 may be involved in chicken liver energy metabolism.

### CRISPR/Cas9 mediating chicken *VNN1* gene knockout

**Figure 2:**
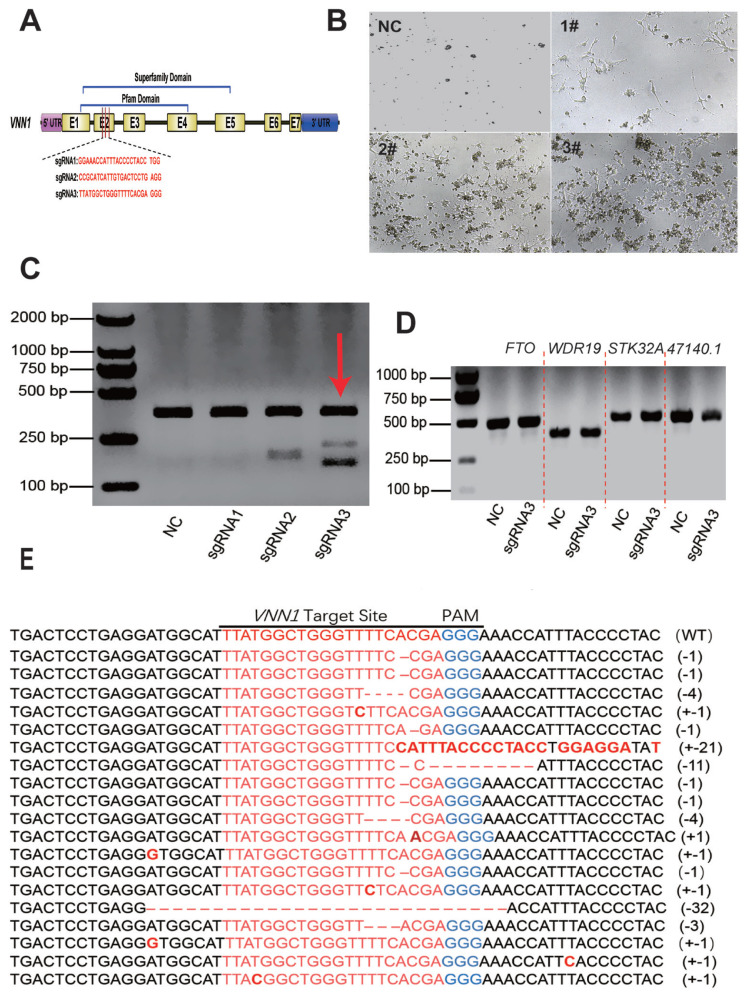
CRISPR/Cas9-mediated *VNN1* gene mutagenesis in chicken LMH cells. We used CRISPR/Cas9 system to edit *VNN1*, according to VNN1 protein domain, the knockout site was designed in second exon, which belong to pfam domain and superfamily domain. (A) A schematic diagram of the structure of the sgRNA targeting region of the chicken *VNN1* gene. Seven coding exons, a 5′ UTR region, a 3′ UTR region and intervening intron sequences are shown with boxes and lines. Three sgRNA-targeting sequences were designed based on the sequence at exon 2. (B) Morphological changes of cells in each group after screening with 2 μg/mL puromycin. Most of the LMH cells died in the NC group and the sgRNA1#-transfected group. More LMH cells transfected with sgRNA2# survived than cells in the control group, but the number of surviving cells was significantly less than that in the sgRNA3#-transfected group. (C) The cleavage activity of CRISPR/sgRNA *in vitro*. The results of the T7EI assay showed that CRISPR/sgRNA3# mediated VNN1 gene knockout in LMH cells. (D) T7E1 assay of four potential off-target effects. (E) Alignment of TA clone sequences. CRISPR/Cas9, clustered regularly interspaced short palindromic repeats (CRISPR)/CRISPR-associated 9; *VNN1*, Vanin-1; UTR, untranslated region; NC, negative control; T7E1, T7 endonuclease I assay.

To examine whether CRISPR-mediated gene-targeted knockout can be applied to the loss of function in chicken LMH cells, the chicken *VNN1* gene, which is associated with liver lipid metabolism, was chosen as the knockout target. According to the conserved domain of the VNN1 protein, three target sites were designed at exon 2 of the *VNN1* coding sequence to knock out the superfamily domain and Pfam domain (Figure 2A). Three knockout plasmids (pX330-*VNN1*-sgRNA1#, pX330-*VNN1*-sgRNA2# and pX330-*VNN1*-sgRNA3#) were transfected into LMH cells. After 48 h of transfection, screening enrichment was performed with 2 μg/mL puromycin. After 48 h puromycin screening, the cell status of each group changed significantly. As shown in Figure 2B, most of the LMH cells were dead in the control group and sgRNA1#-transfected group. In the NC group without px330 transfection, the cells were mostly dead due to the absence of the resistance gene. Also, cell death in the sgRNA1# transfected group may be caused by transfection. Enhanced cell survival was observed in LMH cells transfected with sgRNA2# compared to the control group, but the number of surviving cells was significantly less than in the sgRNA3#-transfected group.

To further assess the constructed CRISPR/gRNA vector activity, the cells were cultured in fresh medium after puromycin selection, and genomic DNA was extracted when the cell confluence reached 90%. We amplified a single product consisting of approximately 540 bp that covered the target site using the primers described in Supplementary Table S1. The PCR products were examined by a T7 endonuclease I (T7E1) assay. The results indicated that sgRNA2# and sgRNA3# had greater cleavage activity than sgRNA1# (Figure 2C). As the sgRNA3# group exhibited two distinct bands in the T7E1 enzyme assay (Figure 2C), we sequenced its target site by cloning the PCR product into the pMD-19T vector. TA clone sequencing showed deletion and insertion at the knockout site, indicating that the *VNN1* was knockout (Figure 2E). In summary, these experimental results indicated that the CRISPR/sgRNA3# site had strong knockout activity.

The off-target effect is the most prominent issue with the CRISPR/Cas9 system. In previous studies, frequent off-target phenomena were observed in CRISPR-mediated mutagenesis in transformed cell lines [22,23]. To test whether the CRISPR/sgRNA3#-mediated knockout effect in this experiment was affected by off-target factors, we performed a T7E1 cleavage assay on four candidate off-target genes based on site prediction. As shown in Figure 2D, none of the four candidate genes with high off-target risk showed off-target phenomena. These results indicated that the CRISPR/Cas9 system guided by the designed *VNN1*-sgRNA3# was highly specific for targeted mutagenesis on the chicken *VNN1* gene.

**Figure 3:**
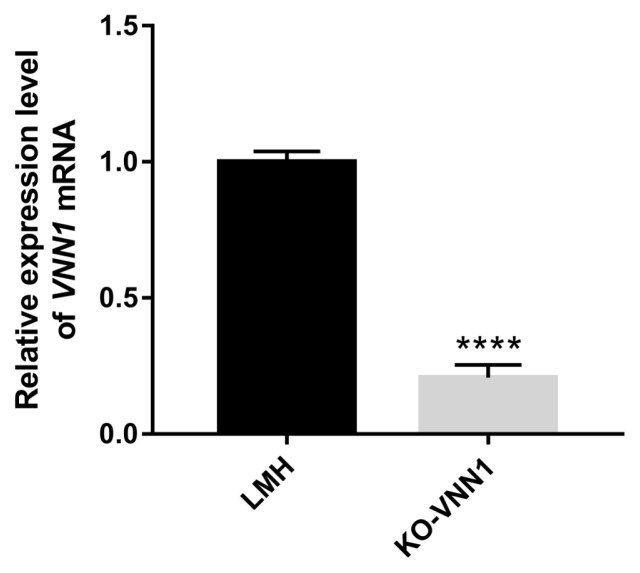
Expression of the *VNN1* gene in wild-type LMH and *VNN1*-modified LMH cells. Validation of *VNN1* knockout. The RT-qPCR results showed that the expression level of *VNN1* mRNA in cells in the CRISPR/sgRNA3# system (KO-*VNN1* group) significantly decreased by 90% compared with that in the wild-type cells (LMH group). The data are reported as the means±standard error of the mean (n = 3). The mRNA gene expression was normalized to a housekeeping gene, β-actin. **** indicates p<0.0001 vs LMH cells (NC). *VNN1*, Vanin-1; RT-qPCR, real-time quantitative polymerase chain reaction; NC, negative control.

A subclone of cells, which were verified to have knockout activity by T7E1 and TA cloning assays, was enriched by multiple rounds of expansion culture-puromycin screening cycles for 12 days. And, a complete selection period requires 3 to 4 days. During the enrichment of *VNN1*-modified LMH cells, we did not find significant differences in cell morphology compared to the wild-type LMH cells. However, we found a small number of cell deaths in the initial screen, possibly from unsuccessfully transfected cells or the loss of intracellular puromycin resistance genes. The RNA of the subclone of cells was extracted, and RT-qPCR was performed to detect the expression levels of *VNN1* mRNA. The results of RT-qPCR showed that the expression level of *VNN1* mRNA in the CRISPR/sgRNA3# system significantly decreased by 90% compared with that in the control group (Figure 3). This result indicated that the subclone of cells with *VNN1* knockout was successfully established.

### Effect on lipid deposition after *VNN1* knockout

**Figure 4:**
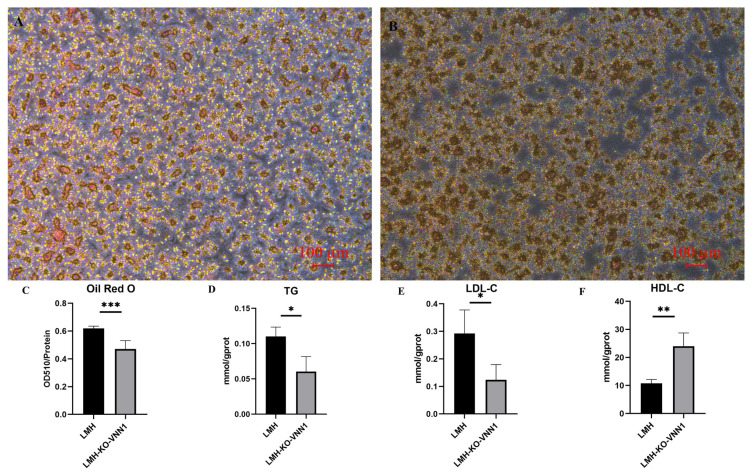
Effect of *VNN1* knockout on lipid deposition in LMH cells. We observed and recorded the changes in lipid deposition after *VNN1* knockout and the results showed that the lipid deposition was changed after *VNN1* knockout. A was control group and B was LMH-KO-*VNN1* cells, recorded at light field, bar: 100 μm. We also quantitatively analyzed the changes of oil red staining in each group of cells. C shows the results of quantitative analysis. To ensure that the two groups of cells were at the same level, the OD value of the cells was corrected by the protein concentration of the cells. Panels D to F are the contents of TG (D), LDL-C (E) and HDL-C (F) measured in LMH-KO-*VNN1* cells and control cells. *VNN1*, Vanin-1; OD, optical density; TG, triglyceride; LDL-C, low-density lipoprotein cholesterol; HDL-C, high-density lipoprotein cholesterol. *** p<0.001, ** p<0.01, and * p<0.05.

To verify the effect of knocking out *VNN1* on lipid deposition in LMH cells, we performed oil red staining and the contents of TG, LDL-C, and HDL-C assays. The results of oil red staining showed that after knocking out *VNN1*, the stained cells were reduced, indicating that lipid deposition in LMH-KO-*VNN1* cells was decreased (Figure 4A and 4B). At the same time, OD510/protein concentration was used as the detection value to quantitatively analyze the reduction of lipid deposition. These results showed that after knocking out *VNN1*, compared to the control group, OD510/protein concentration had a significant 0.7-fold decrease (p<0.001), indicating that lipid deposition decreased after knocking out *VNN1* (Figure 4C). To reveal the effects of *VNN1* knockout on TG, LDL-C, and HDL-C, we detected the contents of TG, LDL-C, and HDL-C in LMH-KO-*VNN1* cells. The results showed that after knocking out *VNN1*, the contents of TG and LDL-C were decreased significantly, which were 0.5 times (p<0.05) and 0.3 times (p<0.05) of the control group, respectively (Figure 4D and 4E). The content of HDL-C was significantly increased by 2.5 times compared with the control group (Figure 4F). These above results further indicated that lipid deposition was changed after *VNN1* knockout.

### Differential expression of mRNAs in LMH cells with *VNN1* knockout

**Figure 5:**
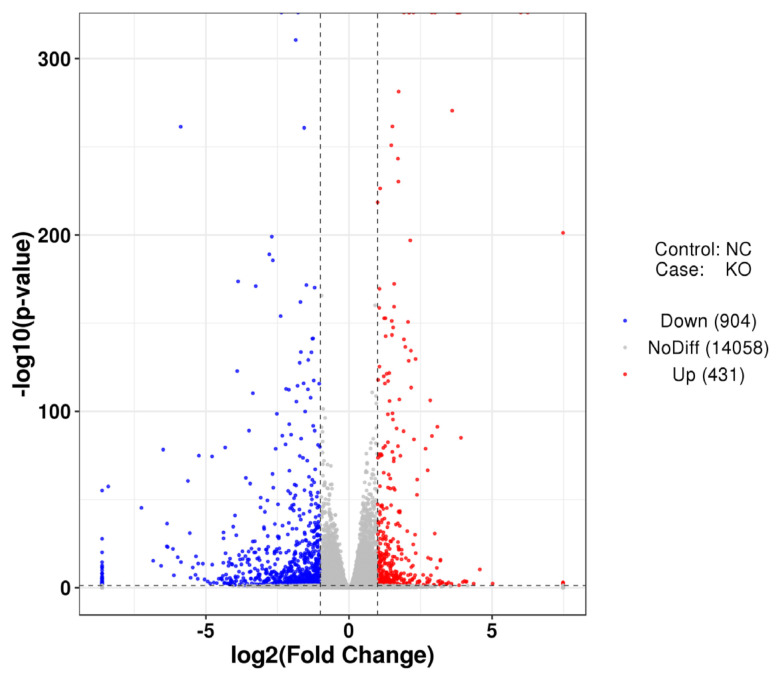
Analysis of differential gene expression after *VNN1* gene knockout. Overview of the differentially expressed genes for each comparison samples. Volcano plot of differentially expressed genes between the KO-*VNN1* group and the control group. The abscissa is log2 fold change, and the ordinate is −log10 (p-value). *VNN1*, Vanin-1.

To better understand the differential expression of intracellular genes caused by defects in the *VNN1* gene in LMH cells, the control group NC and the experimental group KO were compared to identify DEGs. The distribution of differential gene expression is shown in Figure 5. Compared with the control group, 431 genes were upregulated and 904 genes were downregulated after *VNN1* knockout.

### Gene ontology and KEGG pathway analyses of DEGs

**Figure 6:**
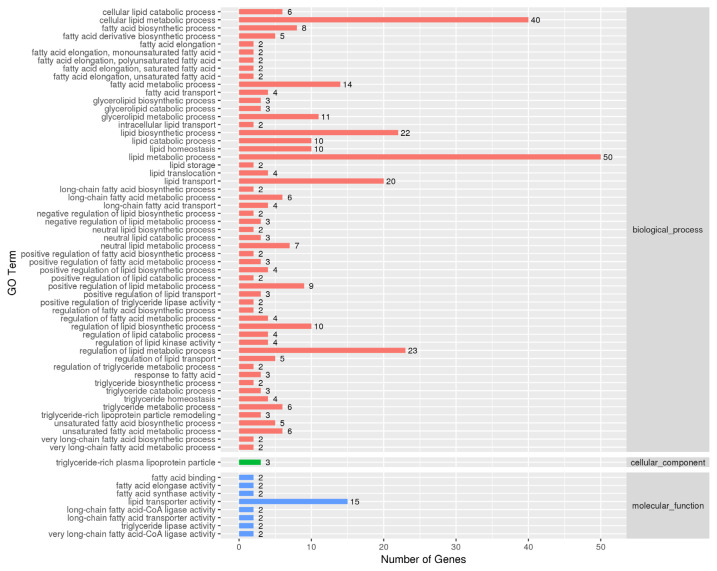
GO analysis of lipid metabolism related DEGs. GO enrichment analysis of differentially expressed genes. This histogram shows the GO terms enriched by all lipid metabolism-related differential genes. Enriched items were measured by the number of differentially expressed genes. GO, gene ontology; DEGs, differentially expressed genes.

**Figure 7:**
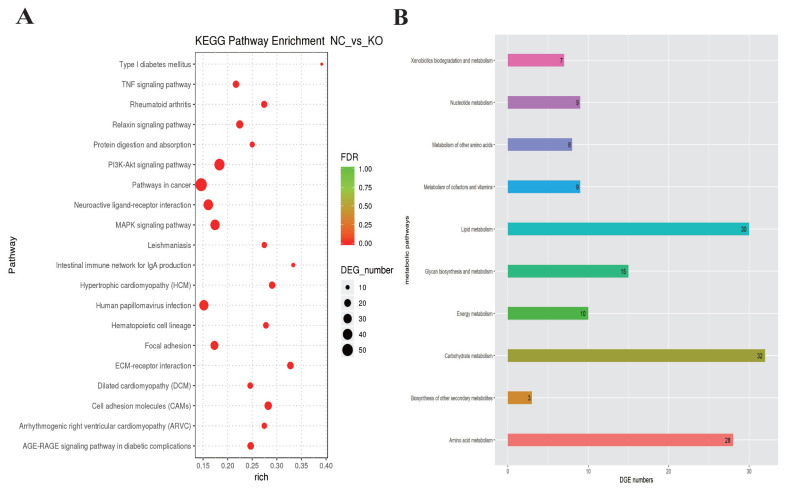
KEGG analysis of lipid metabolism related DEGs. KEGG enrichment analysis of differentially expressed genes. (A) Scatter plots of the top 20 enriched KEGG pathway terms from all DEGs. Enriched items were measured by the rich factor, q value (q<0.05) and the number of genes. (B) This histogram shows the re-clustering analysis of the “metabolism level” in the KEGG enrichment results. Enriched items were measured by the number of DEGs. KEGG, Kyoto encyclopedia of genes and genomes; DEGs, differentially expressed genes.

To understand the functions of the DEGs and the biological processes involved in *VNN1* gene defects in LMH cells, GO enrichment analysis of differential genes (DEGs) was carried out in this study. As shown in Figure 6, several GO terms were enriched for fatty acid metabolism, lipid transport and TG metabolism, such as GO:0042304 (regulation of fatty acid biosynthesis), GO:0019217 (regulation of fatty acid metabolism), GO:0042759 (long chain fatty acid biosynthesis), GO:0006869 (lipid transport), GO:0006641 (TG metabolic process), GO:0070328 (TG homeostasis), and GO:0090207 (regulating the metabolic process of TGs). Then, all the DEGs were mapped to the reference pathway in the KEGG database to identify the metabolic pathways and signal transduction operating in the VNN1-modified LMH cells. The top 20 significantly enriched KEGG terms are displayed in Figure 7A. The enriched signal pathways were mainly related to the “PI3K-Akt signalling pathway”, “MAPK signalling pathway”, “TNF signalling pathway” and “relaxin signalling pathway”. The re-cluster analysis of “metabolism level” in the results of KEGG enrichment showed that “lipid metabolism pathway”, “energy metabolism”, “carbohydrate metabolism” and “glycan biosynthesis and metabolism” were enriched, as shown in Figure 7B. Through the screening of DEGs involved in lipid metabolism-related metabolic pathways in the above GO enrichment pathway and KEGG enrichment pathway, it was found that a total of 76 genes had significant differences, including 29 upregulated genes and 47 downregulated genes. Some of the DEGs are shown in Table 2.

### Validation of selected DEGs by RT-qPCR

**Figure 8:**
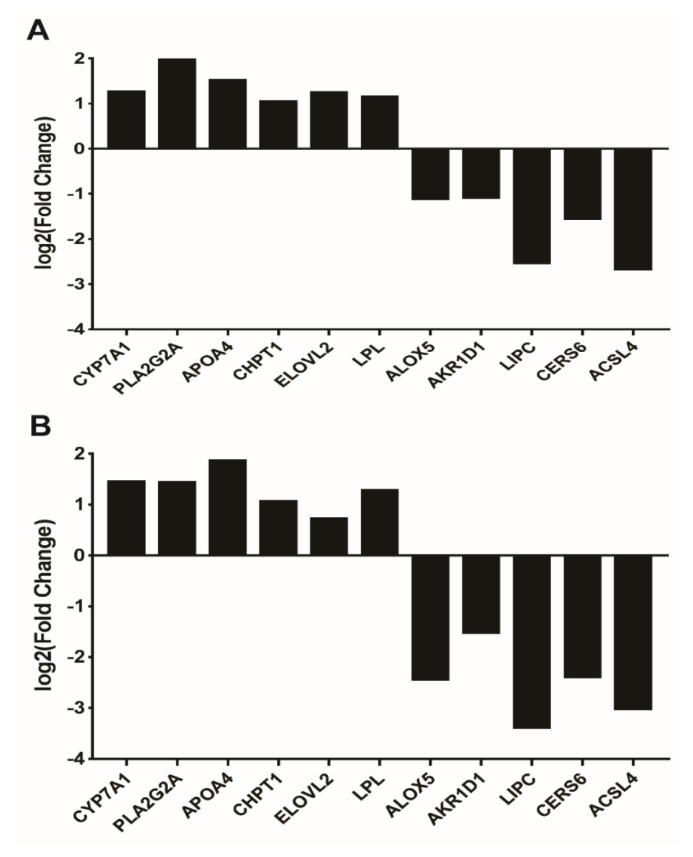
Confirmation of transcriptomics sequencing data by RT-qPCR. Eleven DEGs involved in the phagosome pathway were selected for RT-qPCR. (A) Transcriptome sequencing results of selected lipid metabolism-related DEGs; (B) RT-qPCR verification results of selected lipid metabolism-related DEGs. Log_2_ fold change indicates the log 2 value of the enrichment ratio of each gene expression amount in the experimental group compared with the control group. RT-qPCR, real-time quantitative polymerase chain reaction; DEGs, differentially expressed genes.

To verify the reliability of the sequencing data, 11 DEGs involved in lipid metabolism pathways enriched by GO and KEGG were randomly selected for RT-qPCR analysis. As shown in Figure 8A (transcriptome sequencing data) and Figure 8B (RT-qPCR verification results), the expression levels of 11 DEGs were largely consistent with those of transcriptome sequencing analysis. These results confirm the reliability and accuracy of the transcriptome sequencing data.

## DISCUSSION

The liver is an important metabolic organ that controls energy metabolism and maintains metabolic homeostasis during fasting. Specifically, the liver can coordinate the supply-demand relationship between glycogen and lipids in changing dietary conditions to ensure an adequate supply of energy across tissues. Fasting-refeeding cycles are an important model for studying energy metabolism. A previous study has shown that many genes are regulated in the livers of 4-week-old male chickens during fasting for 16 or 48 h. These altered genes are mainly clustered in glycolipid metabolism (e.g., lipid biosynthesis, TG synthesis, fatty acid beta-oxidation, and gluconeogenesis) and signalling pathways involved in energy metabolism (e.g., insulin signalling pathway, oestrogen receptor signalling pathway and cAMP-mediated signalling pathway) [24]. Consistent with previous findings, this study indicated that genes related to energy metabolism in chicken livers (e.g., *FASN*, *ATGL*, *PPARα*, and PPARG coactivator 1 alpha [*PGC1A*]) showed significant changes in expression after 24 h of fasting, and after refeeding, the expression of these genes was restored at different levels (Figure 1). Notably, the expression of the *VNN1* gene in the chicken liver also increased significantly after 24 h of fasting and returned to the original level after refeeding (Figure 1F). Therefore, we speculated that fasting-induced *VNN1* expression may indicate that *VNN1* is involved in chicken energy metabolism.

Previous studies showed that fasting could promote the accumulation of TG and plasma free fatty acid (FFA) in mice, while knocking down the *VNN1* gene could increase the accumulation of TG in the liver but had no effect on plasma FFA. Further microarray analysis showed that the knockdown of *VNN1* led to the differential expression of hepatic steatosis genes [11]. Moreover, Rommelaere et al [10] also reached a similar conclusion. These findings indicate that *VNN1* is likely a potential novel molecule involved in liver glycolipid metabolism. Therefore, we are particularly interested in the role of *VNN1* in chicken liver lipid metabolism. To investigate the biological function of *VNN1* in chicken lipid metabolic pathways, we tentatively knocked out the *VNN1* gene in chicken LMH cells using the CRISPR/Cas9 system. CRISPR/Cas9 is a newly developed high-efficiency genome editing tool. In the present study, we successfully knocked out the *VNN1* gene in chicken LMH cells, and the TA cloning assay initially estimated that the knockout efficiency was 38.7%. Together with the previously published studies regarding chicken genome editing [17,18,25], the broad applicability of this technology in chicken genome editing was strongly demonstrated. However, most of these studies only indicated that CRISPR/Cas9 can knockout genes in chicken cell lines (mainly chicken DF-1 cell lines), and functional studies of deletion genes have rarely been reported. Therefore, we further studied the role of the *VNN1* gene in liver lipid metabolism based on the targeted knockout of the *VNN1* gene, which also provided favourable evidence for the successful application of CRISPR/Cas9 technology in poultry.

LMH cells require strict conditions for survival, and it is difficult to grow these cells when a small number of cells are present. Therefore, screening monoclonal cell lines is difficult to achieve. In this experiment, after transfecting LMH cells with pX330-sgRNA3#, the edited cell population (named *VNN1*-modified LMH) was enriched by multiple rounds of puromycin screening to obtain more stable knockout cells. RT-qPCR demonstrated that the expression of the *VNN1* gene in *VNN1*-modified LMH cells was extremely significantly (p<0.0001) less than that in wild-type LMH cells. As shown in the RNA-seq data, several GO terms were enriched in fatty acid metabolism, lipid transport and TG metabolism. However, the top 20 significantly enriched metabolic pathways for the DEGs in *VNN1*-modified LMH cells were mainly concentrated in human disease processes. In addition to the metabolic pathways associated with human disease, the re-cluster analysis of “metabolism level” in the results of KEGG enrichment showed that “lipid metabolism pathway”, “energy metabolism”, “carbohydrate metabolism” and “glycan biosynthesis and metabolism” were enriched. We further examined changes in lipid metabolism-related gene expression in *VNN1*-modified LMH cells and found that the expression of genes, such as ELOVL fatty acid elongase 2 (*ELOVL2*) and apolipoprotein A4 (*APOA4*), was significantly upregulated. *ELOVL2* is closely related to the biosynthesis of long-chain fatty acids in chickens [26]. ApoA4, a key apolipoprotein, has been demonstrated to have several proposed roles, including lipid transport, lipid absorption and lipoprotein metabolism [27]. *ELOVL2* and *APOA4* play important roles in lipid transport, synthesis, and deposition. Therefore, we concluded that silencing the *VNN1* gene promotes upregulation of the *ELOVL2* and *APOA4* genes, thereby promoting lipid deposition in chicken liver cells to a certain extent. Notably, the study conducted by Diepen et al [11] in rats also reached a similar conclusion, indicating that the inhibition of Vanin-1 activity in rats induces the accumulation of hepatic TGs upon fasting.

In addition, a clinical study has indicated that *VNN1* gene expression levels and G137T polymorphisms are closely related to HDL-C levels in Mexican-American adults, particularly in prepubescent girls. The expression of *VNN1* is downregulated when the genotype is TT along with a lower level of HDL-C [28]. Hu et al [29] also showed that *VNN1* promotes the accumulation of cholesteryl esters by inhibiting the expression of PPARγ and liver X receptor (LXR) α in THP-1 macrophage-derived foam cells. In the present study, CRISPR/Cas9-mediated knockdown of the *VNN1* gene in LMH cells caused a significant increase in Cholesterol 7α-hydroxylase (*CYP7A1*) gene expression. CYP7A1, a vital rate-limiting enzyme in the bile acid biosynthetic pathway in the liver, can catalyse the initial step in cholesterol catabolism and bile acid synthesis. Overexpression of CYP7A1 can lead to decreased plasma HDL-C levels [30,31]. Therefore, *VNN1* may be involved in the metabolism of cholesterol in chicken liver cells through the *VNN1*-*CYP7A1* pathway. In summary, our results strongly suggest that VNN1 may be a core component in the regulation of lipid metabolism in chicken livers.

In conclusion, our results suggest that *VNN1* is a key component that orchestrates chicken liver lipid metabolic pathways. Additionally, in this study, we successfully used the CRISPR/Cas9 system to efficiently achieve targeted mutations in chicken LMH cells, which provided favourable evidence for the successful application of CRISPR/Cas9 technology in poultry. To the best of our knowledge, this study is the first to employ the CRISPR/Cas9 system to achieve the targeted knockdown of endogenous genes in chicken LMH cells and to investigate the subsequent changes in liver lipid metabolism.

## Figures and Tables

**Table 1:** Nucleotide sequences of sgRNA for the target sites

Name	Oligo nucleotide sequences
VNN1-sgRNA1#	F: 5′-CACCGGAAACCATTTACCCCTACC-3′
	R: 5′-AAACGGTAGGGGTAAATGGTTTCC-3′
VNN1-sgRNA2#	F: 5′-CACCGCCGCATCATTGTGACTCCTG-3′
	R: 5′-AAACCAGGAGTCACAATGATGCGGC-3′
VNN1-sgRNA3#	F: 5′-CACCGTTATGGCTGGGTTTTCACGA-3′
	R: 5′-AAACTCGTGAAAACCCAGCCATAAC-3′

F was the target of each knock site except for the underlined letters, and the underlined letters was the part that must be added during the design of the sgRNA Oligo pair for the construction of the knockout plasmid. “R” was a sequence that is reverse complementary to F.

**Table 2:** Partial lipid metabolism related differentially expressed genes

Gene	NC	KO	Fold change	log_2_(Fold change)	p-value
*CYP7A1*	10.15327728	24.27610302	2.390962282	1.257591371	0.009858937
*PLA2G2A*	3.47383023	14.42975905	4.153846933	2.054448055	0.002955509
*CHPT1*	135.186802	278.9085004	2.063134095	1.044837593	3.62061E-14
*GYS2*	40.39835061	130.2643685	3.224497203	1.689074218	4.35297E-07
*ELOVL2*	195.1841469	461.3213462	2.363518521	1.24093617	3.58984E-17
*LPL*	429.8021314	957.436755	2.227622166	1.155504553	1.49286E-47
*MTTPL*	11.94484203	24.84052871	2.079602949	1.056308106	0.025965028
*LIPI*	139.8111526	298.7771522	2.137005144	1.095590381	9.8118E-09
*APOA4*	355.7152212	1016.541017	2.857738314	1.514873814	9.39517E-78
*AGT*	130.2605656	628.2687483	4.823169201	2.269981422	8.22977E-85
*BDH1A*	153.838343	9.725470051	0.063218765	−3.983503338	9.30105E-42
*PCK1*	341.7386205	48.2790286	0.141274722	−2.823424745	5.62269E-24
*ALDH1A3*	2962.524964	1379.74864	0.465734013	−1.102421847	4.56412E-46
*GALNT9*	443.9340818	75.14942234	0.169280588	−2.562511552	1.82246E-79
*UAP1L1*	722.1625156	336.1283577	0.465446974	−1.103311276	1.11722E-34
*GFPT2*	1005.511735	488.647837	0.485969303	−1.041062907	1.0334E-41
*ACSL4*	1911.128398	301.9975901	0.158020565	−2.661815766	2.5684E-186
*CERS6*	203.4185778	69.82683612	0.34326676	−1.542597934	1.50385E-19
*LIPC*	40.51574093	7.06859714	0.174465454	−2.518986698	1.00499E-08
*FABP4*	7.926794932	1.541152387	0.194423143	−2.362728133	0.030652645
*RARRES2*	1289.790738	469.6317871	0.364114715	−1.457535049	8.75643E-73
*ST8SIA2*	100.2561332	38.2322463	0.38134571	−1.390828625	5.82961E-09

*PLA2G2A*, phospholipase A2 group IIA; *CHPT1*, choline phosphotransferase 1; *GYS2*, glycogen synthase 2; *ELOVL2*, ELOVL fatty acid elongase 2; *LPL*, lipoprotein lipase; *MTTPL*, microsomal triglyceride transfer protein; *LIPI*, lipase I; APOA4, apolipoprotein A4; *AGT*, angiotensinogen; *BDH1A*, 3-hydroxybutyrate dehydrogenase 1A; *PCK1*, phosphoenolpyruvate carboxykinase 1; *ALDH1A3*, aldehyde dehydrogenase 1 family member A3; *GALNT9*, polypeptide N-acetylgalactosaminyltransferase 9; *UAP1L1*, UDP-N-acetylglucosamine pyrophosphorylase 1 like 1; *GFPT2*, glutamine-fructose-6-phosphate transaminase 2; *ACSL4*, acyl-CoA synthetase long chain family member 4; *CERS6*, ceramide synthase 6; *LIPC*, lipase C, hepatic type; *FABP4*, fatty acid binding protein 4; *RARRES2*, retinoic acid receptor responder 2; *ST8SIA2*, ST8 Alpha-N-acetyl-neuraminide alpha-2,8-sialyltransferase 2.

## SUPPLEMENTARY MATERIAL

**Supplementary Figure S1**. pX330 plasmid map.

**Supplementary Figure S2**. Schematic representation of the sgRNA and RT-qPCR primer targeting *VNN1* gene.

**Supplementary Table S1**. All primers used in this study
