# Supplementary figures and images for: CRISPR/Cas9-mediated knockout of the Vanin-1 gene in the Leghorn Male Hepatoma cell line and its effects on lipid metabolism
Source: Anim Biosci. 2023 Nov 1;37(3):437–50. doi: 10.5713/ab.23.0162 (PMC10915194; doi:10.5713/ab.23.0162)

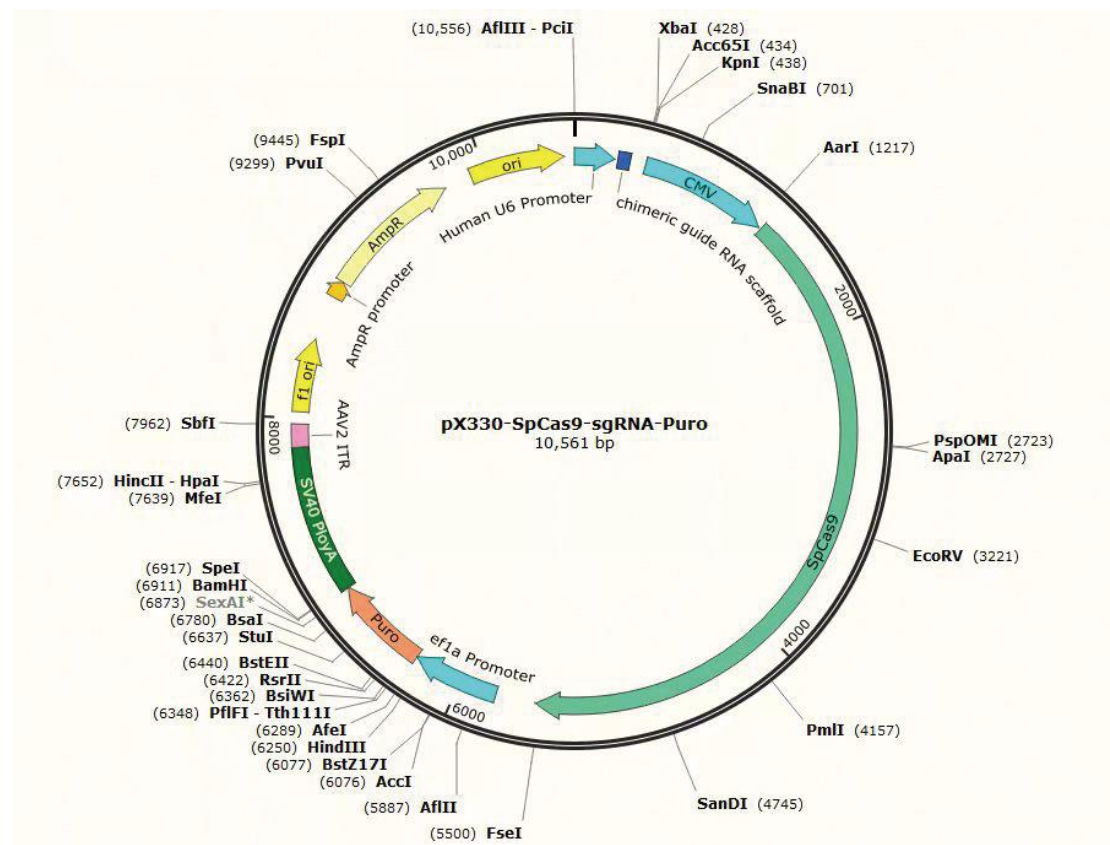

Supplementary Figure S1. pX330 plasmid map

Supplement: Supplementary file 1 [file ab-23-0162-Supplementary-Fig-S1.pdf]
