# Supplementary material for: CRISPR/Cas9-mediated knockout of the Vanin-1 gene in the Leghorn Male Hepatoma cell line and its effects on lipid metabolism
Source: Anim Biosci. 2023 Nov 1;37(3):437–50. doi: 10.5713/ab.23.0162 (PMC10915194; doi:10.5713/ab.23.0162)
Supplement: Supplementary file 2 [file ab-23-0162-Supplementary-Fig-S2.pdf]

Exon1:ATGGTCCCTTCCCAGACCCTGGTGTGTGCTGCAGTGCTTGCCCTGGCAG

Initiation codon

TCCTGCAGGCCCTGGCCTCAGACACCTTCATCGCAGCCGTGTACGAGCACGCCG

TCATCCTGCCACGCACCATTCACAAGGTTTCTCCTGCTGATGCTTTGGCCCTGAT

GAACAGAAACATGGATGTTCTGGAAGGAGCTGTCAAGGAAGCAGCCCAGCAG

Exon2:GGTG*CCCGCATCATTGTGACTCCTG*AGGATGGCAT*TTATGGCTGGGTTTTCAC*

sgRNA2# **sgRNA3#**

*GA*GG*GAAACCATTTACCCCTACC*TGGAGGATATTCCTGATCCAGAGGTGAACTGGA

sgRNA1#

TTCCCTGCACCGATCCCACAAG

Exon3:ATTTGGTCGAACACCAGTGCAGGAACGACTCAGCTGCATGGCCAGGAAC

AACTCCATCTATGTAGTGGCAAATATCGGGGACAAGAAGCCATGTAATTCCAGTG

→ ***VNN1*-qF**

ACCCCAAGTGCCCCAGTGATGGTCGCTACCAGTACAACACTGACATTGTCTTTGA

CTCTGAAGGGAAACTGGTGGCTCGTTACCACAAG

Exon4:TATAATCTGTTTATGTCAGAAGCAAATTATTTTAATTACCCAAAGGAGCCA

GAAGTCATCACCTTCGAGACTCCCTTTGGGAAGTTTGGCATTTTCACCTGCTTTG

***VNN1*-qR** ←

ACATTCTTTTCCGGGAGCCTGCTGTGGTGCTGGTGAGCAAGCTACAGGTGGACA

Supplementary Figure S2. Schematic representation of the sgRNA and RT-qPCR primer targeting *VNN1* gene

The single underline in exon 1 represented the initiation codon ATG, the italic plus single underline in exon 2 was marked with different sgRNA, and the double underline in exon 3 and exon 4 was labeled with *VNN1* fluorescence quantitative primer.
