# Supplementary material for: CRISPR/Cas9-mediated knockout of the Vanin-1 gene in the Leghorn Male Hepatoma cell line and its effects on lipid metabolism
Source: Anim Biosci. 2023 Nov 1;37(3):437–50. doi: 10.5713/ab.23.0162 (PMC10915194; doi:10.5713/ab.23.0162)
Supplement: Supplementary file 3 [file ab-23-0162-Supplementary-Table-S1.pdf]

Supplementary Table S1. All primers used in this study

Primer name	GenBank accession numbers	Primer Sequence (5′–3′)	Primer purpose	Amplicon sizes
gga-VNN1-EXON2-F<br>gga-VNN1-EXON2-R	-	ACAAGGTTTCTCCTGCTGA<br>CCCGATATTTGCCACTACAT	**T7E1** assay<br>TA clone	**296**
VNN1-Off-target-WDR19-F1<br>VNN1-Off-target-WDR19-R1	-	CCCATTAGAAAGAGGCATTG<br>AGACTTACTTGGGAGCCTTG	**off-target analysis**	**378**
VNN1-Off-target-47140.1-F<br>VNN1-Off-target-47140.1-R	-	ACCCACGTTAGCATCGGTAT<br>CCAACCTGTCCCATCTGC	**off-target analysis**	**475**
VNN1-Off-target-FTO-F1<br>VNN1-Off-target-FTO-R1	-	GAAACTGGGAGAAAGCAACT<br>CAACCATACCTCAATCCAAC	**off-target analysis**	**471**
VNN1-Off-target-STK32A-F<br>VNN1-Off-target-STK32A-R	-	TAATAGCATCATACCCAACG<br>ACTTGCGATGCCTTCACT	**off-target analysis**	**487**
gga -VNN1-qF<br>gga -VNN1-qR	NM_001379258.1	GACTCTGAAGGGAAACTGGT<br>CAAAGCAGGTGAAAACGCCA	RT-qPCR	142
gga-β-actin-qF<br>gga-β-actin-qR	NM_205518.1	CACGGTATTGTCACCAACTG<br>ACAGCCTGGATGGCTACATA	RT-qPCR	200
gga-PLA2G12A-qF<br>gga-PLA2G12A -qR	NM_001389517.2	ATCGACGTTTACCTCAACGC<br>CCAAATAGAGGCGATCCGCA	RT-qPCR	146
gga-RACGAP1-qF<br>gga-RACGAP1-qR	XM_004949732.4	CGCCAGGCTGAAGTTTTAAG<br>GCTCAGCTTCACTTTTCATC	RT-qPCR	145
gga-G6PC-qF1<br>gga-G6PC-qR1	XM_040692407.1	TCCAGCAGTTCCCACTCA<br>GAGGAAAGTGAGCAGCGA	RT-qPCR	234
gga-PCK1-QF<br>gga-PCK1-QR	NM_205471.2	CTGCTGGTGTGCCTCTTGTA<br>TTCCCTTGGCTGTCTTTCC	RT-qPCR	259
gga-FASN-qF<br>gga-FASN-qR	NM_205155.4	TCTCTGCCATCTCCCGAA<br>AGCGCCTTCCATTCTCTA	RT-qPCR	187
gga-PPARA-qF<br>gga-PPARA-qR	NM_001001464.1	AGGAGAACCATCCGATTGA<br>CTCAGACCTTGGCATTCGT	RT-qPCR	162
gga-PGC1A-qF<br>gga-PGC1A-qR	NM_001006457.2	CCAGTCTGAGGTCATCTCCA<br>CATTGGTCACATCTCCATCTG	RT-qPCR	179

gga-ACOX1-qF1	NM_001006205.2	TTAATGACCCTGACTTCCAGC	RT-qPCR	160
gga-ACOX1-qR1		CGATGAACAAAGCTTTTAAACCAG		
gga-CPT1A-QF	NM_001012898.1	AACCCTTGACACAACTGGCT	RT-qPCR	96
gga-CPT1A-QR		GTGACGATAAGGGCAACCCA		
gga-ACACA-F1q	NM_205505.2	TTCCTACCAAGACTCCCTAT	RT-qPCR	194
gga-ACACA-R1q		GGTTTCTACGGCAACTACTC		
gga-SCD-QF	NM_204890.2	CAGCGGAGATACTACAAGCCC	RT-qPCR	141
gga-SCD-QR		GGCATTGAGCCCTAAGGTGT		
gga-ATGL-qF1	NM_001113291.2	TCCAAGGAGGAGCTGATC	RT-qPCR	195
gga-ATGL-qR1		ACTGTCTCGTGGGCAGAT		
gga-LPL-QF	NM_205282.2	CGATCCCGAAGCTGAGATGAA	RT-qPCR	169
gga-LPL-QR		CCACCCATGGATCACCACAA		
gga-CYP7A1-qF	NM_001001753.2	AGGAGGCAATGAGGCTATCG	RT-qPCR	171
gga-CYP7A1-qR		TGAGTGTCAAGGGATCAGCA		
gga-PLA2G2A-qF	NM_001277914.1	TCACCTGCGGTGATGAGCAG	RT-qPCR	174
gga-PLA2G2A-qR		AACTGGCCGTGTGTGGATTT		
gga-apoA4-qF	NM_204938.3	CAAGGAGACCGTGGACCAAC	RT-qPCR	160
gga-apoA4-qR		TGAGTCCTGCACCAGCTGTG		
gga-ELOVL2-qF	NM_001197308.2	GAAAGCTTTTGACCAGGAAG	RT-qPCR	154
gga-ELOVL2-qR		GAACTTGTTGCCCAGCCATA		
gga-CHPT1-qF	NM_001030764.2	TGGATAGCATTGTTCATATCCTTGT	RT-qPCR	192
gga-CHPT1-qR		TGCTTCATTCAGCATTCCGC		
gga-ALOX5-qF	XM_040675251.1	CATGCCGTCCTACACCGTTA	RT-qPCR	163
gga-ALOX5-qR		GTAGGAGTCAACCGCGCC		
gga-CERS6- qF	XM_040676746.1	CTTGTGGGTCTTCAACGTGC	RT-qPCR	134
gga-CERS6-qR		TTGTTACATGCAAGGGGTTCC		
gga-LIPC-qF	XM_040680794.1	ACTCTCAAGTCCACTGCGTT	RT-qPCR	184
gga-LIPC-qR		GTGATCCCAGTGCCTCTTTTT		
gga-ACSL4-qF	XM_040670601.1	TGAGCTGAGCTGAGATTTAACTG	RT-qPCR	84
gga-ACSL4-qR		CTTTGAGAGCCAGCAGTGGA		
